# Community-Based Exercise Programs Post Spinal Cord Injury Hospitalization: A Pilot Study for a Randomized, Multicenter, Double-Blind Controlled Setting

**DOI:** 10.3390/life14091135

**Published:** 2024-09-09

**Authors:** Dongheon Kang, Jiyoung Park

**Affiliations:** 1 Department of Healthcare and Public Health Research, National Rehabilitation Center, Ministry of Health and Welfare, Seoul 01022, Republic of Korea; jakekang@korea.kr; 2 Department of Safety and Health, Wonkwang University, Iksan 54538, Republic of Korea

**Keywords:** rehabilitation, spinal cord injuries, physical fitness, community health service, intervention

## Abstract

This study explores the effectiveness of community-based exercise programs for individuals with spinal cord injury (SCI) following hospital discharge. Given the rising incidence of SCI, particularly in South Korea, and the associated long-term disabilities, the necessity for comprehensive post-discharge rehabilitation is paramount. The study focuses on a pilot randomized multicenter double-blind controlled trial, targeting SCI patients who have completed inpatient rehabilitation and are living in the community. The primary aim is to evaluate the impact of structured exercise programs on physical fitness, functional capacity, and overall recovery. The research introduces the SpinalFit program, a community-based intervention designed to enhance muscle strength, cardiopulmonary endurance, and mobility through a combination of aerobic and resistance training. This program addresses the critical need for safe and effective rehabilitation options outside the hospital setting, utilizing circuit training with resistance bands and body-weight exercises tailored to each participant’s capacity. The study also investigates barriers to physical activity in the community for SCI patients and the potential role of community exercise centers in bridging this gap. Preliminary findings from this pilot trial are expected to provide valuable insights into optimizing exercise regimens for SCI patients, informing future large-scale studies and contributing to improved post-discharge rehabilitation strategies.

## 1. Introduction

Over the past few decades, there has been an increase in the incidence of spinal cord injury (SCI), establishing it as a major contributor to disability on a global scale [1,2]. SCI is a major health issue with substantial impacts on morbidity and long-term disability in many countries, including the Republic of Korea [3,4]. In South Korea, approximately 2500 new cases of SCI are reported each year, placing a significant burden on the healthcare system and society [5]. Recent improvements in initial treatments and follow-up care for SCI have resulted in higher survival rates, emphasizing the necessity for comprehensive individuals with SCI management after hospital discharge [3,6,7,8].

Damage to the spinal cord results in significant muscle degeneration and loss of strength, which severely restricts and impairs physical abilities. Decreased muscle strength is linked to reduced endurance capacity. Furthermore, a reduction in cardiopulmonary endurance usually arises, adversely impacting self-reliance and general well-being [9,10,11,12]. Numerous studies have shown that greater muscle strength and lean mass are associated with lower all-cause mortality rates in healthy individuals and those with chronic conditions [13,14,15,16,17,18]. Poor cardiopulmonary endurance is a strong predictor of both overall death rate and the risk of heart and vascular conditions, irrespective of age and other contributing variables [19,20,21,22,23]. Reduced cardiopulmonary endurance is also associated with a higher risk of SCI complications [24,25]. Therefore, cardiopulmonary endurance is an important factor for predicting the overall health and recovery outcomes in individuals with SCI [26].

The beneficial impact of exercise training on post-discharge recovery highlights the importance of integrating structured physical activity into the rehabilitation process for individuals with spinal cord injury (SCI) [27]. Exercise training, characterized by planned, organized, and repetitive physical activities, is essential for enhancing or maintaining functional capacities [28]. Ensuring that individuals with SCI participate in exercise training is critical, as insufficient activity levels are linked to an increased risk of secondary health issues [29]. To promote successful physical activity participation and improve outcomes such as cardiorespiratory fitness, functional capacity, and mobility, it is vital to develop and implement carefully tailored exercise programs for SCI patients [26]. Extensive meta-analyses have demonstrated the significant influence of exercise parameters—such as intensity, frequency, type, duration, and volume—on rehabilitation outcomes, consistently showing that well-designed exercise regimens can substantially enhance recovery in SCI patients [24,30,31,32,33,34,35,36].

Individuals with SCI often face significant functional challenges that necessitate comprehensive rehabilitation. Recently, exercise-based approaches have gained attention due to their ability to enhance physical fitness [26,37]. Engaging in both aerobic and resistance exercises can help to alleviate the disabilities associated with SCI through multiple physiological pathways. Combining aerobic and resistance training is particularly beneficial, as it effectively improves muscle strength, cardiorespiratory health, and overall functional capacity. Aerobic exercises primarily enhance cardiovascular fitness and mobility, while resistance training supplements these benefits by increasing muscular strength and physical performance [38,39,40,41]. Systematic reviews and meta-analyses suggest that integrating both types of exercise into rehabilitation protocols yields the best outcomes for SCI patients [24]. Despite these advantages, many individuals with SCI remain physically inactive or engage in minimal physical activity post discharge, which hampers their recovery [11,27,42]. Furthermore, participation in exercise programs is often restricted in community settings due to safety concerns, highlighting the need for innovative strategies to ensure safe and effective exercise regimens [30,43]. The exercise protocol employed in this study aligns with internationally recognized guidelines, such as those established by the American College of Sports Medicine (ACSM), for physical activity and rehabilitation in individuals with spinal cord injury. By adhering to these standards, our study aims to extend the application of these guidelines to a community-based setting for SCI rehabilitation in South Korea. One potential solution is to facilitate access to community exercise centers under medical supervision, ensuring that patients receive appropriate guidance and support.

After hospital discharge, it is essential to assess the effectiveness of exercise training programs for individuals with SCI, taking into account their overall health, physical fitness, muscular strength and muscle function, cardiorespiratory endurance, and physical composition, as well as their medical history and physician’s guidance. Despite the importance of such evaluations, there is a lack of research in South Korea focusing on SCI survivors who have been medically prescribed to participate in exercise programs at community fitness centers to ensure safe and effective rehabilitation post discharge. To address this gap, we have launched a collaborative initiative called SpinalFit, which is a holistic fitness program aimed at improving the physical health and quality of life of individuals with SCI. This program brings together experts in rehabilitation and exercise science to evaluate the impact of community-based exercise interventions prescribed by doctors for SCI patients after their release from the hospital. Therefore, this research focuses on assessing the impact of community-based exercise training tailored for individuals with SCI after hospital discharge. While the broader SpinalFit program focuses on comprehensive long-term rehabilitation, this paper specifically investigates the early post-discharge phase, assessing the impact of structured exercise on physical recovery and functional capacity.

## 2. Methods and Materials

### 2.1. Research Framework

This pilot research is structured as a double-blind, randomized, multicenter trial with parallel groups targeting individuals with their first SCI. Individuals with SCI will be evenly and randomly distributed between the control and experimental groups. To ensure confidentiality, the evaluators conducting subsequent assessments will remain blind to the group assignments. The study will take place at four separate hospitals in Korea: the Korea National Rehabilitation Hospital, Korea University Anam Hospital, Bucheon Sports Medicine Hospital, and Korea National Health Insurance Service Hospital in Ilsan.

**Figure 1:**
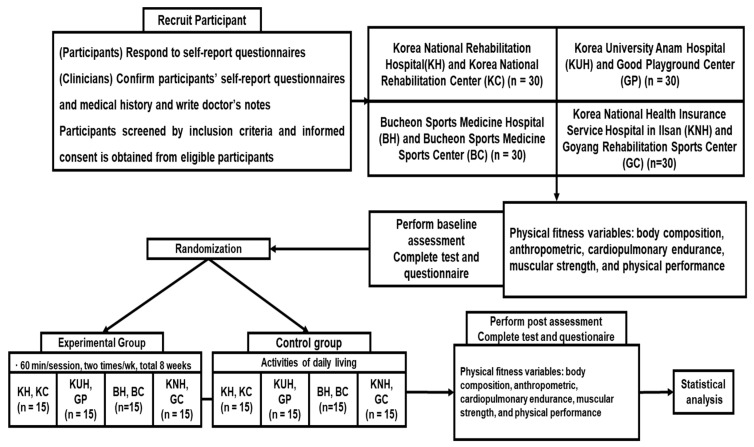
Flowchart.

Individuals with SCI will be registered in the SCI rehabilitation programs offered at the participating hospitals, which serve both inpatient and outpatient populations. Throughout the study, an investigator who will not know the participants’ group assignments will conduct evaluations with the participants’ consent. The study will be conducted in collaboration with the National Rehabilitation Hospital, with the research protocol developed under their guidance. The Institutional Review Board of the National Rehabilitation Hospital has granted ethical approval for this research (NRC043052021). The study protocol has been registered under the identifier KCT0007521. This protocol complies with the SPIRIT guidelines [44]. Figure 1 presents a flowchart detailing the study design.

### 2.2. Participants

The study targets adults aged 18 years and older. This age criterion was selected to ensure consistent physiological responses to the exercise intervention, given the varying effects of exercise across different age groups. Additionally, participants will need to have been discharged from the hospital for at least 6 months, ensuring that they have reached a stable baseline in their recovery, allowing the intervention to focus on enhancing their physical capabilities rather than managing acute recovery phases.

To be included in the study, individuals will need to have a documented diagnosis of SCI, have completed their early medical treatment and recovery, currently live in the community, and have provided an explicit agreement to participate. Participants will be excluded if they are under inpatient care at the time of the study, are unable to engage in physical activities due to other neurological conditions (aside from SCI), are experiencing orthopedic issues in the lower limbs or significant heart or lung problems, or if they are determined by the assessor to be incapable of carrying out the required study activities.

Participants’ prior sporting experience will be recorded during the baseline assessment and considered during the randomization process. Stratified randomization will ensure that differences in previous exercise experience are balanced between the experimental and control groups, thereby minimizing potential biases in the study outcomes.

### 2.3. Blinding and Randomization

Once informed consent has been obtained, the team supervisors will select and refer suitable participants for the study. The randomization process will then be conducted by an independent statistician who is not part of the research team. To ensure balanced allocation, participants from each institution will be randomly assigned to one of two subgroups using a stratified block allocation technique, with block sizes of two per stratum, achieving an equal distribution between the exercise (EXG) and the control group (CNG).

This allocation sequence is set to be generated using version 23.0 of SPSS software. The block size and seed number will be randomly determined by the statistician responsible for the process. The resulting group assignments will be placed in opaque envelopes, each labeled for a specific individual, and given to the study manager at the onset of the treatment phase.

This research will utilize a double-blind approach, ensuring that both the participants and the evaluators remain unaware of the group assignments. Additionally, the instructors responsible for delivering the interventions will remain unaware of the group assignments for the duration of the study to minimize any possible bias and maintain the reliability of the outcomes, ultimately reinforcing the trustworthiness of the conclusions. Nevertheless, it is important to acknowledge that the principal investigator, due to the trial’s design, will have access to the group assignments.

To maintain blinding during the assessments, evaluators will receive only coded data with no indication of group assignments. Evaluators responsible for measuring physical fitness and cardiopulmonary outcomes will be separate from those managing participant enrollment and randomization, thereby minimizing the risk of bias. Additionally, to further enhance blinding integrity, data analysis will be performed independently by statisticians unaware of the participant groups.

### 2.4. Procedure

During the first appointment, the research staff will assess prospective candidates to ascertain their suitability based on the predefined eligibility requirements. The investigators will thoroughly explain the study, present the consent forms, and discuss the details with the participants to ensure that informed consent is obtained. Written informed consent will be gathered at this point. Afterwards, participants will fill out a self-administered questionnaire, which they will submit to a doctor for a medical evaluation and review of their responses. Before the allocation process, experienced evaluators will assess the outcome measures for each SCI.

### 2.5. Intervention Program

The intervention program was specifically designed for individuals with SCI and consists of a combination of physical rehabilitation exercises to improve muscle strength, flexibility, and functional mobility. The exercises include resistance training, functional movement drills, and aerobic activities tailored to the needs and abilities of SCI patients.

Each exercise will be carefully selected and adjusted according to the participant’s functional capacity and ASIA impairment level. For instance, participants with severe motor impairments will perform modified versions of resistance exercises, such as supported trunk movements and assisted stretches, ensuring safety and effective engagement. The progression model will be dynamically tailored to each participant’s condition, adjusting resistance levels and repetitions to match individual capabilities while maintaining a balance between challenge and safety.

The program will be delivered in a group setting, where participants will engage in structured exercise sessions together. However, each exercise routine will be personalized to the individual’s capacity, allowing for tailored modifications within the group environment. This approach combines the benefits of group dynamics with the necessity for individualized training, ensuring that all participants progress safely and effectively.

To accommodate varying degrees of impairment, the intervention protocol will provide detailed guidelines for alternative exercises according to each ASIA impairment level. These modifications will enable all participants to engage in the program without risking injury or exacerbation of their condition. Safety and progression will be continuously monitored by certified fitness instructors, who will adjust exercise intensity based on real-time heart rate tracking and participant feedback. This careful monitoring will ensure that each participant advances within safe limits, particularly when integrating higher resistance levels or more complex movements during the later stages of the program.

The exercise regimen will consist of 1 h sessions occurring twice weekly over an 8-week period, as specified in Table 1. The program will include three circuits, each consisting of four to seven exercises that integrate both resistance and aerobic training. Individuals will complete one set of movements, alternating between upper and trunk exercises to minimize fatigue, with rest intervals of 1–2 min between each set. The circuit training combines resistance and aerobic exercises performed at a range of 65 to 80 percent of the participant’s peak heart rate determined during the initial assessment. Individuals will be instructed to perform training sessions at a moderate intensity, 12 to 13 on the scale [45].

Resistance training will utilize TheraBands targeting the arms, shoulders, and back (e.g., lateral raise, chest press, lat pulldown, triceps extension) and trunk exercises (e.g., seated twist, back extension, deadlift). The color of the band will indicate the resistance level, and intensity will be assessed using the TheraBand Resistance Exercise Perceived Exertion Scale [46]. Participants will determine the appropriate band color by performing lateral raises at 15 maximum repetitions (RM) [46,47]. They will then complete three sets of 12–15 repetitions for each exercise, following a power training protocol [48] involving rapid concentric contractions, a 1 s pause, and a slow eccentric contraction phase lasting over 2 s.

Aerobic exercises primarily involve non-equipment-based activities. During the initial two weeks, participants will perform body-weight exercises to reduce injury risk and acclimate to the regimen. From weeks 3 to 5, resistance bands will be incorporated into aerobic routines, with exercises at 15 RM. Starting from week 6, participants will progress to higher resistance bands. For individuals with limited use of the paretic side, a supportive hand wrap will be provided to help fasten the band while exercising. An instructor will customize the endurance training intensity to match each participant’s specific conditions.

Participants in the CNG will receive standard care, which will include general health advice, routine medical follow-ups, and access to community support services. Although they will not engage in structured exercise programs, they will be encouraged to maintain regular activities of their daily lives. The control group’s care will mirror typical post-discharge practices in South Korea, without the addition of targeted physical exercise interventions. This standard care approach ensures that any differences observed between groups can be attributed to the specific exercise regimen provided in the experimental group.

Certified fitness instructors will supervise the program, tracking participants’ peak heart rates with a heart rate monitor and an iPad. The intensity and difficulty of exercises will be progressively increased by adding more repetitions and sets, ensuring gradual overload and consistent progress.

Participants will be required to attend at least 10 out of the 16 total sessions. Considering that the study will involve both inpatients and outpatients, alternative arrangements, such as make-up sessions, will be available for those facing scheduling conflicts or health-related issues, ensuring consistent participation.

Given the pilot nature of this study, potential side effects such as increased pain, fatigue, or falls will be closely monitored throughout the intervention. Participants will be regularly assessed for adverse reactions, and the program will be adjusted as needed. Any participant reporting significant discomfort will receive modified exercises to mitigate risks while continuing to benefit from the intervention.

### 2.6. Measurements

The selected outcome measures focus on key areas critical to SCI rehabilitation, including muscular strength, cardiopulmonary endurance, and functional capacity. These metrics are well-established indicators of recovery in SCI patients and align with the study’s objectives of improving physical fitness and mobility. The outcome measures include assessments of muscular strength and function, cardiopulmonary endurance, functional capacity, and physical composition, which together determine overall physical fitness. These metrics are tailored to spinal cord injuries, enabling a detailed evaluation of the participants’ recovery journey and improvements in functionality after the training.

To ensure consistency across the four study centers, all evaluators underwent standardized training, and regular equipment calibration was conducted. Detailed protocols for administering each measurement were provided to ensure uniform procedures across centers, minimizing variability between sites.

Initial measurements and follow-up evaluations will be performed after participants complete the 16 intervention sessions, utilizing the outcome measures listed (Table 2). Additionally, a specially customized questionnaire will be employed to gather sociodemographic and informational data.

#### 2.6.1. Muscular Strength and Function

To assess muscle strength in the upper limbs, grip strength will be measured for each participant with a handheld force gauge. Participants will assume a seated position, with their shoulders slightly adducted and aligned in a neutral posture, with elbows bent at a 90-degree angle, and forearms and wrists held neutrally. Both hands will undergo three test trials following a practice session, and the average of the three readings for each hand will be used in the analysis [48].

#### 2.6.2. Cardiopulmonary Endurance

Cardiopulmonary endurance will be assessed through a graded exercise test to determine peak oxygen uptake for all participants. The test, conducted with an arm cycle ergometer, will commence with a workload of 10 W for the first 2 min, followed by an increase of 10 W at 2 min intervals until the individual voluntarily achieves fatigue. The highest VO2, or endurance capacity, will be recorded when the participant is no longer able to sustain a cadence of 60 RPM [49]. A gas exchange system will analyze expired air for oxygen and carbon dioxide concentrations every 10 s, collected through a facemask that will cover the nose and mouth. Heart rate will be continuously tracked with a Polar monitor, and exertion levels will be measured using the scale of Borg.

A digital spirometer will be used to evaluate lung function [50]. Prior to the tests, participants will receive thorough instructions and demonstrations to ensure accurate results [50]. The evaluation will include measurements of vital capacity, forced expiratory volume in 1 s, inspiratory capacity, expiratory reserve volume, peak expiratory flow, inspiratory reserve volume, and forced vital capacity. In addition, respiratory muscle strength will be determined by measuring maximum inspiratory and expiratory pressure with the Pony FX device [50].

#### 2.6.3. Functional Capacity

To evaluate functional capacity, the Functional Reach Test will be employed [51]. Participants will sit in a wheelchair with an upright posture and reach forward, extending their arms to their maximum reach. A starting position will be measured at the fingertips when the arms are raised to shoulder level. The furthest position reached and maintained for 3–5 s will be recorded as the final measurement. The distance separating the two positions (in cm) will be documented. A staff member will be positioned nearby to offer guidance. Participants will undergo three attempts, with the mean result utilized for evaluation.

#### 2.6.4. Physical Composition

Physical composition will be evaluated through two different methods: whole-body dual-energy X-ray absorptiometry (DXA) with the Discovery Wi system and bioelectrical impedance analysis with the S10 InBody device. DXA will generate detailed data on overall and regional lean body mass as well as body fat. In contrast, the bioelectrical impedance analysis method will assess parameters such as body composition.

### 2.7. Participant Timeline

The timeline for participant recruitment, experimental procedures, and subject evaluations is comprehensively outlined in Table 3, following the SPIRIT guidelines [44].

### 2.8. Procedures for Data Collection

Outcome measures, excluding descriptive and sociodemographic variables, will be gathered both prior to and following the 16 intervention sessions.

### 2.9. Data Analysis

The sample size for this pilot study was determined using G*Power software (v3.1.2) [52], with an effect size of 0.5, an alpha level of 0.05, and a power of 0.8 (80%) to reduce the likelihood of Type II errors. These parameters were selected based on previous studies involving similar community-based interventions for SCI patients. The calculated sample size is 29 participants per group, with an additional participant added to each group to account for potential dropouts, resulting in a total of 120 participants across four centers. Given the pilot nature of this study, the relatively small sample size serves to test feasibility while providing preliminary insights into the program’s effectiveness.

To compare the exercise and control groups, we will analyze participant characteristics and baseline data. A summary table will display the characteristics of individuals across the groups. Continuous variables will be described using averages, standard deviations, middle values, and the range between the 25th and 75th percentiles. This approach guarantees that the groups will be comparable at the start of the study.

Data analysis will follow a strategy that includes all participants as originally allocated. To evaluate the distribution of the primary outcome dependent variables, the Kolmogorov–Smirnov test will be used. These variables will be represented using either averages with standard deviations or middle values with ranges between the 25th and 75th percentiles, depending on their distribution. Hypothesis tests will be conducted for each outcome to fulfill the objective, positing that the outcomes in the EXG will surpass those in the CNG. All estimates are to include intervals with a 95% certainty. For normally distributed independent samples, we will apply Student’s *t*-test, whereas the Mann–Whitney U test will be used for non-normally distributed data.

In evaluating secondary outcomes, hypotheses related to quality of life will be assessed, and estimates will be provided with 95% confidence intervals. A regression analysis will be conducted to explore potential relationships among outcome measures, such as physical fitness measures, including muscular strength, cardiopulmonary endurance, and functional capacity. The strengths of the relationships will be assessed through individual t-tests and F-tests from analysis of variance. The results will be presented as odds ratios with a ninety-five percent confidence interval.

## 3. Discussion

This research will be the first to evaluate the impact of exercise regimens for SCI patients in the Republic of Korea after their hospital discharge and referral to community fitness centers. The implementation of community-based exercise training for SCI patients post-discharge aligns with best practice guidelines [53,54].

The primary physical impairments caused by SCI include muscle atrophy, weakness, loss of muscular strength, decline in cardiopulmonary endurance, reduced functional capacity, and altered physical composition [55]. Maintaining physical fitness in these areas is crucial for daily activities such as transitioning from a wheelchair and turning independently [56]. Consequently, enhancing physical fitness through rehabilitation after SCI is crucial for minimizing nervous system disability during the recovery process [57].

Previous research has demonstrated the benefits of exercise programs in the rehabilitation of SCI patients. Notably, a combination of strengthening and aerobic exercises has been found to enhance the exercise capacity of SCI patients [58]. Recent findings indicate that a combination of aerobic and resistance exercises provides greater benefits to SCI patients compared to aerobic exercises alone [59]. The exercise program used in this study, which incorporates both aerobic and resistance training, aims to improve muscle strength, physical function, cardiorespiratory fitness, and daily life activities [24,37]. By combining these exercise modalities, the program is expected to enhance real-life functions more effectively than interventions targeting individual functions.

A systematic review of combined strengthening and aerobic training for SCI revealed that endurance exercises are mainly carried out using treadmill and cycling machines, while strength training generally involves machine-based methods [37]. However, machine-based exercises carry a risk of injury [40]. To mitigate this risk, our study will incorporate resistance bands and body-weight exercises, focusing on strength and cardiovascular conditioning, to ensure participant safety and reduce the likelihood of harm at local rehabilitation exercise training centers.

Our study suggests that training within community settings will be practical, enabling individuals with SCI to acquire and perform exercises in different locations after hospital discharge and during visits to local rehabilitation exercise training centers. We also expect that participants will find the exercise training enjoyable and beneficial for their recovery. Customizing the exercise regimen based on comprehensive patient data—including SCI classification, disability severity, medical background, and clinical evaluations—will help to identify and mitigate potential risks.

The findings of this study may strengthen the link between individuals with SCI and local rehabilitation exercise training centers after discharge from hospital in Korea. The results of this research will be disseminated through articles in pertinent academic journals and presented at several conferences dedicated to SCI and local healthcare.

However, the study is limited by the small number of participants in each group (15 in the EXG) which, while adequate for a pilot study, may reduce the likelihood of observing meaningful variations between EXG and CNG.

In order to extend this research to larger studies, scaling up the community-based exercise programs for individuals with SCI would require addressing several key challenges. Firstly, maintaining high adherence rates across diverse community settings may require enhanced support mechanisms, such as tailored feedback and progress monitoring, to ensure sustained participation. In addition, providing adequate training for local fitness instructors and ensuring access to appropriate rehabilitation equipment would be essential to replicating the program on a larger scale. Solutions may include collaborations with regional healthcare centers to develop standardized protocols, offering online training modules for instructors, and securing funding to equip community fitness centers with accessible exercise tools.

Moreover, there would likely be logistical challenges related to participant recruitment and data collection in different regions. Developing partnerships with local healthcare providers could facilitate smoother participant enrollment, and adopting mobile health technology could streamline data collection processes. By implementing these strategies, future large-scale studies could better evaluate the effectiveness of such interventions across varied demographics and locations, ensuring broader applicability and the sustainability of the program.

Building on this pilot research, we intend to design a comprehensive study to evaluate the impact of community-based exercise training on physical outcomes such as muscular strength, cardiopulmonary endurance, and functional capacity. The initial outcomes will provide valuable insights into critical factors such as sample size, exercise training duration, session length, weekly frequency, control interventions, and assessment. Guidance on incorporating exercise facilities into post-hospital care will be vital for shaping future research initiatives. Our research team is currently developing a collaborative initiative that brings together experts in rehabilitation and physical training to support SCI in exercise programs post hospital discharge. Our long-term goal is to develop an exercise training module that complements our community-based programs, enhancing accessibility and effectiveness.

The findings from this pilot study will have important implications for the development of scalable community-based exercise programs for SCI patients. The positive adherence rates and initial outcomes suggest that such programs can be successfully implemented in diverse community settings, provided that adequate support and tailored modifications are offered. When comparing our findings with similar studies conducted both locally and internationally, the integrated approach of combining aerobic and resistance exercises appears to yield superior outcomes compared to single-modality interventions. However, it is important to acknowledge potential limitations such as self-reported biases in adherence tracking and the logistical challenges associated with maintaining high participation levels in community-based settings. These factors will need careful consideration in the design of future large-scale studies.

## 4. Conclusions

The outcomes of this study could provide valuable understanding of the impact of training regimens for individuals with SCI who are discharged with a doctor’s recommendation and referred to local rehabilitation exercise training centers.

These results might enhance our understanding of how effectively SCI patients can engage in physical activity within a community setting after being discharged from the hospital. In addition, if this innovative training approach proves successful, it may provide crucial insights for creating more comprehensive guidelines for individuals with SCI going forward.

## Figures and Tables

**Table 1:** Intervention protocol.

Warm-Up/Cool-Down		Resistance–Upper Extremities	Resistance–Trunk	Aerobic
Aerobic	Flexibility			
Freewheeling	Static stretching Dynamic stretching	Upper Body Shoulder Press Lateral raise Front raise Bent-over lateral raise Chest Press Chest fly Lat pulldown Bent-over row Back row Biceps Curl Triceps Extension Triceps kickback	Overhead side bend Overhead twist Overhead bend Seated forward press Seated twist Bend and twist Side bend Back extension Seated cat camel Deadlift	Seated Walking on the spot without moving forward Shuttle run

**Table 2:** Data collection involves gathering medical records and anthropometric measurements.

Medical Part	Age	Years
	Gender	Male or female
	American Spinal Injury Association (ASIA) level	A, B, C, D, E
	Hypertension, anemia, dyspnea or asthma, orthostatic hypotension, diabetes, medications for heart disease, coronary stent, epilepsy, medications for anticoagulants, medications for depression, rigidity, autonomic dysreflexia, bladder management, bowel care, pressure ulcer, acute low back pain within 4 weeks, joint pain, medications for osteoporosis, hip or femur fractures	Yes or no
Anthropometric Part	Blood pressure	mmHg
	Height	cm
	Weight	kg
	Body mass index	Underweight/normal weight/overweight
	Days of discharge from the hospital	Number of days

**Table 3:** Study duration: schedule for participant enrollment, interventions, and evaluations.

Study Period				
	Enrollment	Allocation	Post-Allocation	Closeout
Timepoint, baseline	t	0	T16	tx
Enrollment				
Eligibility screening	X			
Informed consent	X			
(Participant) Self-report questionnaires	X			
(Clinicians) Confirm participants’ self-report questionnaires and medical history and write doctor’s notes	X			
Allocation		X		
Assessments				
Baseline variables		X		
Post-intervention variables			X	X
Intervention				
Experimental group			X	X

## Data Availability

Upon reasonable request, the data will be provided by the authors.
